# Risk Factors of Dystocia in Nulliparous Women

**Published:** 2014-05

**Authors:** Rahele Alijahan, Masoumeh Kordi

**Affiliations:** 1 Province Health Center, Ardebil, Iran;; 2 Department of Midwifery, School of Nursing and Midwifery, Mashhad University of Medical Sciences, Mashhad, Iran

**Keywords:** Dystocia, Risk factor, Iran

## Abstract

**Background: **Detection of women at risk for dystocia will allow physicians to make preparations and treatment decisions that can minimize maternal and neonatal morbidity. We aimed to determine the risk factors for dystocia in nulliparous women.

**Methods:** This case series enrolled 447 nulliparous women who presented with a single pregnancy in the vertex presentation and gestational age of 38-42 weeks. Maternal anthropometric measurements were obtained upon admission. We defined dystocia as a cesarean section or vacuum delivery for abnormal progression of labor as evidenced by the presence of effective uterine contractions, cervical dilation of less than 1 cm/h in the active phase for 2 h, duration of the second stage beyond 2 h, or fetal head descent less than 1 cm/h. Data were analyzed by SPSS software version 11.5. Kruskal-Wallis, logistic regression, chi-square, Student’s *t *test and the Mann-Whitney tests were used as appropriated.

**Results:** The state anxiety score (OR=10.58, CI: 1.97-56.0), posterior head position (OR=9.53, CI: 4.68-19.36), fetal head swelling in the second stage of labor (OR=6.85, CI: 2.60-18.01), transverse diagonal of Michaelis sacral ≤9.6 cm (OR=6.19, CI: 2.49-15.40), and height to fundal ratio <4.7 (OR=2.68, CI: 1.09-10.60) were significant risk factors for dystocia.

**Conclusion: **Critical care during labor and delivery in women who have a height to fundal height ratio of <4.7 or transverse diagonal of Michaelis sacral ≤9.6 cm, an anxiety score greater than moderate, and posterior head position or fetal head swelling during the second phase could play an effective and important role in preventing dystocia.

## Introduction

Dystocia is defined as slow progression or lack of progression of labor which occurs in 25-30% of nulliparous women and is regarded as the cause for two thirds of cesarean sections in these women.^1^^,^^2^ If not diagnosed and treated, this condition can lead to maternal/fetal morbidity and even the mother’s mortality.^3^^-^^5^ Women who experience dystocia often undergo surgical interventions such as emergency cesareans, and vacuum and forceps deliveries which cause considerable physical problems for mothers, in addition to stress and an economic burden on the family and community.^6^ Identifying women at risk for dystocia prepares physicians for on time treatment and enables them to minimize maternal-fetal trauma that accompanies this midwifery emergency.^7^ Therefore, one of the main objectives of pregnancy care is the identification of high risk women for dystocia.^8^ In this direction, numerous investigators have attempted to find indexes to identify high risk women during pregnancy. A number of researchers have regarded factors such as mother’s age, height, weight before pregnancy, body mass index (BMI), weight gain during pregnancy, fundal height, birth weight, and foot length of the mother as risk factors. These factors, however, are controversial.^9^

Surapanthapisit and Thitadilok have shown no significant differences between two groups in terms of maternal height (P=0.77). However, age (P<0.05) and weight before pregnancy, BMI, weight at the end of pregnancy, weight gain during pregnancy, fundal height and birth weight (P<0.001) were more in the dystocia group.^10^ In a study by Van Bogaret, foot length measurement (P<0.001) and lower limb length (P<0.014) in the dystocia group was less whereas vertebral length showed no difference between the two groups.^11^ Kirchengast and Hartmann found no significant relationship between weight before pregnancy and BMI to mode of delivery.^12^ Chittithavorn and Pinjaroan observed no significant relationship between mother’s age, height and birth weight with mode of delivery.^13^ In a study by Barnhard et al., women with height to fundal high ratios <3.7 experienced seven times more cesarean sections.^14^ Despite numerous efforts in this field to identify risk factors for dystocia, there is little advancement, hence it is necessary to conduct additional research.^15^ This study aims to determine the risk factors for dystocia in nulliparous women. Most studies have been conducted in countries with different lifestyles, nutritional status and race. To date, no study has been conducted in Iran in this field. Therefore, we intend to identify risk factors for dystocia in nulliparous women.

## Materials and Methods

We conducted this case series study on 525 nulliparous women who referred to the Maternity Department at Omolbanin Hospital, Mashhad, Iran. Their gestational age was ≥38 weeks with single birth and cephalic presentation. The women were introduced from December 2009 until June 2010. Mashhad University of Medical Sciences (MUMS) Ethics Committee approved this project and the ethical aspects were considered for all subjects. Sample size was calculated based on the results of a pilot study with a confidence interval of 99% and maximum standard error of 0.01 by the proportion ration formula. The study continued until 56 women who had dystocia were included. This number of samples was obtained after the participation of 447 women in the study. Of these, 391 had natural delivery and were considered as the control group. We excluded the remaining 78 women who underwent cesarean sections for conditions other than dystocia such as thick meconium-stained amniotic fluid, fetal heart rate deceleration, placenta abruption, severe hemorrhage, non-response of ineffective uterine contractions to oxytocin, and birth weight of <2500 g. Maternal anthropometric measurements at admission and during cervical dilation of ≤5 cm were measured by a researcher. Mother’s weight was measured using a plate scale and her foot length by a wooden centimeter. Head circumference (distance between most prominent part of the occipital bone and middle of the forehead), vertebral length as distance between first cervical spine to the end of the sacrum, length of lower limb length for right side, distance between greater trochanter to the heel, Michaelis sacral transverse diameter (distance between two depressions of superior posterior spines at two horizontal ends of the sacral bone) and vertical diameter of Michaelis sacral (distance between L5 and S1 5^th^ lumbar spine and last sacral spine) were measured using a centimeter tape measure with the mother in the standing position.

Maternal height was measured in the standing position following standards of measuring height; fundal height and abdominal circumference were measured by a centimeter tape measure in the supine position. Mother’s weight before pregnancy or at the first trimester was retrieved from the mother’s prenatal care records and the BMI was calculated. State and trait anxiety at admission were measured using Spielberger’s State-Trait Anxiety Questionnaire which is a standard 40-item questionnaire. In this study, mothers experienced pain and lacked adequate concentration to read and answer questions. Thus, the questions and answers were read by the researcher and the mothers selected the appropriate answers. In this study head circumference to height ratio was divided by 100, and height to fundal height was also calculated; data related to labor and delivery were collected by continuous control of mother during labor and delivery. A researcher managed the delivery by performing hourly examinations of dilation, effacement, and fetal head descent. Patients were considered to have met the criteria for dystocia if, despite the presence of an effective contraction during active labor the rate of cervical dilation was less than 1 cm for 2 h, or during the second phase the rate of fetal head descent was less than 1 cm/h, or if the duration of the second phase was more than 2 h.^1^^,^^2^^,^^16^ After delivery, subjects were divided into two groups of normal delivery (n=391) and dystocia (n=56). If there were 3 uterine contractions in 10 min of 40 s duration or more and no penetration of the finger to the uterine fundus in palpation, patients were considered to have effective contractions.

The validity of the questionnaire was confirmed by content validity and the skill of the researcher for measuring Michaelis sacral vertical and transverse diameters, foot length, vertebral length and length of the lower extremity was confirmed by a three session educational course in the presence of an anatomist. The reliability of the questionnaire was confirmed as r≥0.84 and inter-rater reliability was used for confirming the researcher’s performance in measuring Michaelis sacral vertical and transverse diameters, height, and other measurement criteria for this process. First, they were measured in ten nulliparous females by the researcher and an anatomist. The correlation coefficient was calculated and confirmed as r≥0.84. Inter-rater reliability was used to control the uterine contraction (r=0.943). The reliability of the centimeter tape was confirmed by a wooden centimeter. Data were analyzed by SPSS 11.5 using the Mann-Whitney test for the relationship between quantitative variables without normal distribution, the student’s *t* test for quantitative variables with normal distribution, chi-square for the relationship between quantitative nominal variables, and the Kruskal-Wallis test for the relationship between qualitative rating variables. Logistic regression was used for determining the odds ratio of the variable with a significant difference between both groups. Level of significance was considered at P<0.05.

## Results

Of the 525 pregnant women, 78 were excluded due to cesarean sections related to factors other than dystocia. This study was conducted on 447 subjects, 12.1% (n=56) of which had dystocia. The mother’s body features that included height (P<0.001), foot length (P=0.023) vertebral length (P=0.008), length of lower extremity (P=0.001), sacral Michaelis transverse diameter (P<0.001), fundal height (P=0.021), height to fundal height ratio (P=0.001), and head circumference (P=0.040) were significantly lower than the normal delivery group. Mean maternal head circumference to height in the dystocia group was significantly larger (P=0.012). The dystocia group were older (P<0.001). No significant difference was found for BMI, sacral Michaelis vertical diameter and abdominal circumference (table 1).

**Table 1:** Distribution of maternal age and anthropometric measurements according to delivery method

**Variables **		**Normal delivery** **mean±SD** **n (%)**	**Dystocia** **mean±SD** **n (%)**	**P value**
Maternal age (years)	19≥	72 (18.4)	3 (5.4)	P<0.001*
	20-24	261 (66.8)	34 (60.7)	
	25-30	51 (13.0)	16 (28.6)	
	30<	7 (1.8)	3 (5.4)	
Body mass index (BMI; kg/m^ 2 ^)	26≥	336 (83.9)	47 (83.9)	0.994^a^
	26<	63 (16.1)	9 (16.1)	
Head circumference (cm)		54.7±2.2	54.4±1.4	0.040^aa^
Foot length (cm)		23.5±1.1	23.0±1.4	0.023^aa^
Vertebral length (cm)		58.5±3.3	57.3±2.7	0.008^aa^
Lower limb length (cm)		79.7±3.7	77.9±4.4	0.001^aa^
Transverse diagonal of Michaelis sacral (cm)		10.3±0.7	9.7±0.9	0.000^aa^
Vertical diagonal of Michaelis sacral (cm)		9.4±0.9	9.6±1.0	0.733^aa^
Fundal height (cm)		32.6±2.4	33.4±2.5	0.021^aa^
Abdominal circumference (cm)		98.6±6.6	99.9±8.2	0.363**
Head circumference to height ratio		34.6±1.5	35.1±1.4	0.012**
Height to fundal height ratio		4.8±0.39	4.6±0.41	0.001**
Height (cm)		158.3±5.3	155.6±6.4	P<0.001^aa^

*Kruskal–Wallis; ^a^Chi-square; ^aa^Mann-Whitney; **Independent sample *t* test

Among the variables related to labor and delivery, a significant difference was found between the groups for fetal head station -3 at admission (P<0.001). Transverse and posterior occipital position in the second stage of labor was higher in women who had dystocia (P<0.001). Fetal head swelling in the second phase of labor (P<0.001) and mean state of anxiety (P=0.030) in the dystocia group were significantly higher, however there was no significant difference between the two groups in terms of anxiety trait (table 2).

**Table 2:** Labor and deliver characteristics in nulliparous women according to mode of delivery

**Variables **		**Normal delivery** **mean±SD** **n (%)**	**Dystocia** **mean±SD** **n (%)**	**P value **
Fetal head position at admission time	Occiput anterior	201 (51.4)	22 (39.3)	0.134^a^
	Occiput transvers	17 (4.3)	3 (5.4)	
	Occiput posterior	173 (44.2)	31 (55.4)	
Fetal head station at admission time	-3	51 (13.0)	16 (28.6)	P<0.001^*^
	-2	171 (43.7)	28 (50.0)	
	-1	150 (38.4)	11 (19.6)	
	0	17 (4.3)	1 (1.8)	
	+1	2 (0.5)	0 (0.0)	
Fetal head position at second stage of labor	Occiput anterior	370 (93.7)	30 (53.6)	P<0.001^a^
	Occiput transverse	21 (5.4)	7 (12.5)	
	Occiput posterior	0 (0.0)	19 (33.9)	
Cervical dilation at admission time		3.2±1.5	2.9±1.6	0.190^aa^
Fetal head swelling at second stage	Yes	114 (29.2)	34 (60.7)	P<0.001^a^
	No	227 (70.8)	22 (39.3)	
Anxiety state		48.7±9.6	52.6±9.8	0.003^**^
Anxiety trait		44.4±7.8	46.7±8.5	0.059^aa^

*Kruskal–Wallis; ^a^Chi-square; ^aa^Mann-Whitney; **Independent sample *t* test

The mean neonatal weight (P=0.009), neonatal head circumference (P<0.001), and chest circumference (P=0.001) in the dystocia group was significantly higher. The mean of the first and fifth APGAR scores were significantly lower in the dystocia group. Fetal sex and gestational age were not associated with dystocia (table 3).

**Table 3:** Neonatal outcomes and characteristics of the study groups

**Variables **		**Normal delivery** **mean±SD** **n (%)**	**Dystocia** **mean±SD** **n (%)**	**P value**
Neonatal weight (kg)		3168.5±354.5	3301.2±346.5	0.009**
Neonatal head circumference (cm)		34.4±1.5	35.2±1.3	P<0.001^aa^
Neonatal chest circumference (cm)		33.1±1.6	33.9±1.6	0.001^aa^
Gestational age (weeks)		39.6±1.1	39.5±1.1	0.475^aa^
Sex of neonate	Female	198 (50.6)	29 (51.8)	0.873^a^
	Male	193 (49.4)	27 (48.2)	
APGAR score	1 min	8.7±0.6	8.4±0.8	P<0.001^aa^
	5 min	9.4±0.6	9.2±0.6	0.024^aa^

^a^Chi-square; ^aa^Mann-Whitney; **Independent sample *t *test

According to the results of stepwise logistic regression analyses, the odds of dystocia significantly increased with conditions such as state anxiety score more than moderate, fetal head position in the occipital posterior position during the second phase of labor, swollen fetal head in the second phase of labor, sacral Michaelis transverse diameter ≤9.6 cm, and height to fundal height ratio <4.7 (table 4). As a adverse outcome of dystocia, a low APGAR score in the first minute was four times higher in the dystocia group (OR=4.04, CI: 1.54-10.60, P=0.005).

**Table 4:** Analysis of risk factors of dystocia using odds ratio by stepwise logistic regression

**Variables **	**Normal delivery** **n (%)**	**Dystocia** **n (%)**	**Odds ratio**	**95% CI**	**P value**
State of anxiety score upper than middle or <43	291 (74.4)	55 (84.1)	10.50	1.97-56.0	0.006
Posterior occiput in second stage of labor	3 (0.8)	23 (43.4)	9.53	4.68-19.36	P<0.001
Fetal head swelling in second stage	114 (29.2)	34 (60.7)	6.85	2.60-18.01	P<0.001
Transverse diagonal of Michaelis sacral <9.6 cm	62 (15.9)	51 (63%)	6.19	2.49-15.40	P<0.001
Height to fundal height ratio <4.7	96 (33.6)	20 (55.6)	2.68	1.09-6.55	0.030

## Discussion

The present study examined the association between maternal anthropometric measurements, neonates, and labor characteristics with dystocia in order to identify the risk factors related to this problem. According to logistic regression analysis, a moderate to high anxiety score at admission was the most important risk factor for dystocia in nulliparous women. Women with moderate to high anxiety score experienced dystocia 10.5 times more than other women. Researchers have concluded that anxiety and fear lead to the production of stress hormones in the body that can interfere with normal delivery and conduct dystocia.^17^ Anxiety causes the release of catecholamines.^18^ Catecholamines, particularly epinephrine, interrupt the coordination of uterine contractions by binding to beta-adrenergic receptors located on the myometrium which slows the progression of labor.^19^ A study by Laursen et al. reported that the cesarean section rate in women who experienced fear of delivery during the third trimester was 1.3 times higher. In this research, mothers exhibited symptoms of increased fear as the time to delivery became nearer.^17^

The second risk factor for dystocia was posterior fetal head position. Women with occipito-posterior fetal head position during second phase experienced 9.5 times more dystocia. Abnormal fetal head positions result in the fetal head introduced with larger diameters and the presence of cephalopelvic disproportion.^20^ An abnormal fetal head position is often related to the type of pelvis. The presence of an android pelvis often causes either resistant transverse arrest or occipital posterior position. The platypelloid pelvis conducts fetal head transverse arrest.^2^ A contracted midpelvis is a common cause for the occipito-posterior position or transverse arrest of the fetal head transverse arrest. In most cases these conditions lead to dystocia.^1^

Before full dilation in prolonged deliveries, a part of fetal head skin located on cervix becomes swollen. The incidence of swelling when the fetal head is located in the lower part of the canal is higher because the outlet provides a source of resistance to the fetal head which most likely occurs in the posterior occipital position and with cephalopelvic disproportion.^1^^,^^21^ According to the results of the current study, women who have experienced 6.8 times more dystocia had fetuses whose heads were swollen.

In the present study women with transverse diagonal of the Michaelis sacral that was ≤9.6 cm experienced 6.1 times more dystocia. The Michaelis sacral is a rhombic space in the sacral bone. The upper angle is located between L5-S1 and the lower angle is consistent with the tip of the coccyx, the lateral angles are at the level of the superior posterior spins.^22^ Initially, Michael proposed the importance of this space for evaluation of pelvic capacity in 1851.^8^^,^^23^ The transverse diameter of this rhombus could be observed between cavities of superior posterior spines on the skin.^23^ According to a number of studies, an abnormal shape and size of the Michaelis sacral rhomboid area indicates an abnormal shape and size of the mother’s pelvis. The results of the present study have supported these findings.

We observed that women with height to fundal height ratings of ≤4.7 experienced 2.6 times more dystocia. Logistic regression analyses showed that maternal height and neonatal birth weight were not significant risk factors for dystocia by themselves. It could be concluded that a normal delivery could be possible despite the shortness of height or macrosomia. If height was in proportion to fetal size, the mother could experience normal delivery; a disproportionate fetal size to the mother’s height was more important in dystocia. Barnhard et al. observed a significantly lower mean height to fundal height ratio in the dystocia group compared to the normal delivery group (P=0.002),^14^ which was consistent with the result of the present study.

In the present study despite a lower mean for mother’s head circumference and higher rate of head circumference to height in the dystocia group according to logistic regression, these findings were not effective on dystocia. The higher ratio of head circumference to height in the dystocia group could be related to the shorter stature of women in this group. The only study in this field was conducted by Connolly et al. who noted opposite findings. These researchers reported higher mean head circumference in the dystocia group. They concluded that larger head circumference was a risk factor for dystocia. In their study the mean ratio of head circumference to height in the dystocia group was higher than the group with normal delivery.^24^

In the present study the mean values for foot and vertebral lengths in the dystocia group were lower. Van Bogaert showed significantly lower mean lower limb (P=0.004), vertebral (P=0.003), and foot (P=0.005) lengths in the dystocia group, which supported the results of the current study.^11^ In the study by Awonuga et al., foot length in the dystocia group was lower (P=0.001).^25^ Rozen Holc et al. reported that the mean foot length in the dystocia group was 21.4 whereas in the normal delivery group it was 22.9 cm (P≤0.001).^23^ Okewole et al. found no significant relationship between foot length and type of delivery (P=0.24).^26^ With regards to the shorter stature of women in the dystocia group in the present study, possibly the lower mean foot and vertebral lengths could be related to the small body size of this group.

As an adverse outcome of dystocia, neonates with dystocia had four times greater first minute APGAR scores <9. Tsvieli et al. reported lower mean first and fifth minute APGAR scores in the dystocia group (P≤0.001).^27^ A number of studies such as the study by Surapanthapisit and Thitadilok have reported no significant relationship between APGAR score and delivery type.^10^ Currently researchers believe that anxiety leads to hyperactive contractions, which inhibit fetal blood supply and result in hypoxia.^17^ In this regard, anxiety is an important risk factor for dystocia, hence the low first minute APGAR score may have been attributed not only to a prolonged delivery but also to the mother’s anxiety.

## Conclusion

According to the results of this study the most important risk factor for dystocia in nulliparous women were moderate to high anxiety scores, occipito-posterior fetal head position, fetal head swelling during the second phase, Michaelis sacral transverse diameter ≤9.6 cm, and height to fundal height ratio <4.7. Measuring these parameters in addition to special care during labor and delivery in high risk women might effectively prevent dystocia and its complications. 
